# Vasculitis associated with familial Mediterranean fever: a study on 16 french adult cases

**DOI:** 10.1186/1546-0096-13-S1-P128

**Published:** 2015-09-28

**Authors:** S Abbara, O Fain, D Saadoun, C Bachmeyer, A Mekininan, K Stankovic Stojanovic, L Mouthon, L Gilardin, S Amselem, G Grateau, S Georgin-Lavialle

**Affiliations:** 1 AP-HP Tenon hospital, Internal Medicin, Paris, France; 2 AP-HP St Antoine hospital, Internal Medicin, Paris, France; 3 AP-HP Pitié-Salpétrière hospital, Internal Medicin, Paris, France; 4 AP-HP Cochin hospital, Internal Medicin, Paris, France; 5 AP-HP Trousseau hospital, Genetics, PARIS, France

## Background

Familial Mediterranean Fever (FMF) has been described in association with various vasculitis, especially Henoch-Schonlein purpura (HSP), polyarteritis nodosa (PAN), protracted febrile myalgia (PFM) and Behcet disease (BD).

## Objectives

To describe the features of French patients with FMF displaying a vasculitis.

## Materials and methods

A multicentric retrospective study was performed thru the French national center of FMF to identify patients displaying a vasculitis and FMF.

## Results

16 patients (12H, 4F) with a median age of 41 years [29-61] were included. Patients were Sefarad Jews (n=9), Turkish (n=2) and Arabic (n=5). Seven of them had a familial history of FMF, none had a familial history of auto-immune diseases. Their FMF was symptomatic during childhood except for two patients; most of them had colchicine. They displayed various type of vasculitis such as: HSP (n=7), PAN (n=4; two of them with viral B hepatitis), Granulomatosis with Polyangitis (GPA) (n=1), Microscopic Polyangitis (MPA) (n=1) and unclassified (n=3). Their vasculitis was diagnosed at a median age of 32 [5-43]. For one patient the diagnosis of vasculitis was made before the diagnosis of FMF. Patients with PAN mostly displayed weight loss, fever, myalgia, abdominal pain, arthralgia, with evidence of vasculitis of small or medium-sized blood vessels on a skin biopsy (n=3) or renal arterial aneurysms on an angiography (n=1). *MEFV* sequencing showed: homozygous M694V/M694V mutations (n=2), homozygous E148Q/E148Q mutation (n=1) and double heterozygous M694V/V726A mutation (n=1).

The patient with GPA displayed an intra-alveolar hemorrhage with ORL involvement. He had a M694V/M694V mutation and was the only patient which FMF was diagnosed after the vasculitis. All of 5 patients with HSP displayed a M694V/M694V *MEFV* mutation.

The patient with MPA displayed erythema nodosum, arthro-myalgia, neuropathy, constrictive pericarditis, and cutaneous vasculitis. He displayed a M694V/E148Q *MEFV* mutation.

## Conclusion

FMF can be associated with various vasculitis, especially PAN and HSP. The most common *MEFV* mutation in our patients is M694V/M694V. This cohort of 16 patients includes various origins such as jewish, Arabic and Turkish. Interestingly, the diagnosis of vasculitis can either precede the diagnosis of FMF, or occur in the course of a previously known FMF. Thus, persistence of an inflammatory syndrome after resolution of vasculitis among a mediterranean patient should lead to look for autoinflammatory disease such as FMF. Actually the link between these two conditions is not known and would need further physiopathological studies.

